# Internet addiction and sleep quality among adolescents in a peri-urban setting in Nepal: A cross-sectional school-based survey

**DOI:** 10.1371/journal.pone.0246940

**Published:** 2021-02-18

**Authors:** Kshitij Karki, Devendra Raj Singh, Dikshya Maharjan, Sushmita K. C., Shreesha Shrestha, Deependra Kaji Thapa

**Affiliations:** 1 Department of Public Health, Asian College for Advance Studies, Purbanchal University, Lalitpur, Nepal; 2 Group for Technical Assistance, Program and Research, Lalitpur, Nepal; 3 Southeast Asia Development Actions Network (SADAN), Innovation, Research and Program, Lalitpur, Nepal; 4 Faculty of Humanities and Social Sciences, Tribhuvan University, Kathmandu, Nepal; 5 School of Health Sciences, College of Health and Medicine, University of Tasmania, Sydney, NSW, Australia; Charite Medical University Berlin, GERMANY

## Abstract

**Background:**

Excessive use of the internet among adolescents often led to later bedtimes and poor sleep quality. This study assessed the relationship between internet addiction and sleep quality among adolescents in a peri-urban setting in Nepal.

**Method:**

This cross-sectional study employed a questionnaire survey among 390 adolescent students recruited from two schools in Kirtipur Municipality in Nepal. The Internet Addiction Test and the Pittsburgh Sleep Quality Index were used to assess internet addiction and poor sleep quality respectively. The association between internet addiction and sleep quality was analysed by logistic regression.

**Results:**

One-fifth (21.5%) of the participants were identified with borderline internet addiction and 13.3% with possible internet addiction. Poor sleep quality was found among 31% of the participants. Internet addiction was significantly associated with poor sleep quality (OR = 1.85, p = 0.022 for borderline, and OR = 3.98, p = <0.001 for possible internet addiction compared to no internet addiction).

**Conclusion:**

Adolescents with internet addiction were more vulnerable to suffer from poor sleep quality. It is recommended that municipalities and schools should aware the adolescent students on the adverse effects of excessive internet use including poor sleep quality. The findings have implications for adolescents, parents, school authorities and researchers.

## Background

The number of people using the internet has surged in the last few years and the internet has become a fundamental part of daily life for many of us. The proportion of the global population using the internet increased from 17% in 2005 to 53.6% in 2019, with more than half of the internet users living in Asia [1]. While the internet penetration is high in developed countries (86.6%), compared to developing (47.0%) and the least developed countries (19.1%), a steady increase can be observed in low-income countries like Nepal in recent years. A recent survey in Nepal reported 51.1% of the households had access to the internet, with 44.2% of the population using it at least once a week [2]. In Kathmandu valley, about 70% of the households had access to the internet [3].

Adolescents and young adults are more likely to use the internet for a wide range of activities such as information search, social networking, communicating, music playing, emailing, playing games and fulfilling their social needs [4]. Increased internet availability with widespread devices such as mobile phones and tablets provide adolescents access to and use of the internet during daytime as well as bedtime. The accessibility of such facilities in developing countries has put adolescents at risk of internet addiction [5–7]. Internet addiction among adolescents have been reported to be associated with poor outcomes including poor mental [8, 9] and physical [10, 11] health, substance abuse [12], academic difficulties [13], social isolation [14], self-injurious behavior and suicidal tendencies [15–17] and low self-esteem [9, 18].

Internet addiction is also reported to be associated with poor sleep quality and sleep disturbances [19–23]. Adolescents who used the internet go to bed later in the night, needed more time to fall asleep and had an increased number of awakenings in the night [24]. 16.5% of Nepalese undergraduate students [25] and 23.8% of Indian medical students [26] internet users had poor sleep quality which led to depressive symptoms.

Although it is a growing concern, data has been limited in Nepal. Various researches have been conducted on internet addiction, and sleep quality and mental health problems in different countries. However, very less evidence is generated in relation to internet addiction, poor sleep quality and related areas among adolescent students in Nepal. The study will help to explore evidence on sleep quality, internet addiction, and its contributing factors. The purpose of this study was to assess the prevalence of sleep quality on the internet using adolescents in a peri-urban setting of Nepal.

## Method

### Study design and setting

The study consisted of a cross-sectional school-based survey conducted among adolescents at Kirtipur municipality in Kathmandu, Nepal. The municipality is located 5 km southwest of Kathmandu city and is a peri-urban setting of Nepal. With the increase in internet facilities coupled with access to smartphones, computers, and laptops in recent years, adolescents and youths using the internet have been significantly increased in peri-urban areas [27]. The study followed the STROBE statement for a cross-sectional study [28].

### Participants’ recruitment and data collection

Two secondary level schools were randomly selected from 37 schools in the study municipality. The number of schools was defined based on the sample size calculated for selecting the students. A total of 390 school students of grades 9 and 10 were selected and all students present at the time of survey completed the questionnaires. The samples were calculated applying the population proportion formula. All students from the selected grades meeting the eligibility criteria were included in the sample through the enumerative sampling technique. The selected students had access to at least one device with internet. Permission from the respective schools was requested by sending a letter to the principal. The objective, process and confidentiality of the study were explained and a consent form was signed by both the participants and their parents. Students were surveyed using a self-administered questionnaire in the classroom setting. This study was approved by the Ethical Review Board of Nepal Health Research Council (Reg. no. 688/2018). The recruitment and data collection were conducted between May to September 2019.

### Measures

#### Internet addiction.

Internet Addiction Test is a validated instrument to measure internet addiction [29]. It consists of 18 items that can be rated in terms of a three-point Likert rating scale (never or rarely, sometimes and often). The scoring ranges from 0 to 36 where 0–14 is considered as no internet addiction, 15–19 as borderline internet addiction, 20–29 as possible internet addiction and ≥ 30 scores as likely internet addiction. Cronbach’s alpha coefficient of the Internet Addiction Test in this study was 0.83.

#### Sleep quality

Pittsburgh Sleep Quality Index is a self-report questionnaire that assesses sleep quality over a month time interval. The measure consists of 19 individual items, creating seven components that produce one global score ranging from 0 to 13. Scores between 0–5 were considered as ‘good sleep quality’ and ≥ 6 as ‘poor sleep quality’ [30]. The Cronbach’s alpha for sleep quality index was 0.71.

Both the Internet Addiction Test and the Pittsburgh Sleep Quality Index scales were translated into the local (Nepali) language with the help of a bilingual (Nepali and English) social scientist. The two public health researchers had performed the face validity of the translated scales. Then, it was back-translated into English by the independent translator confirming the original meaning of the scales and any discrepancies seen were addressed.

#### Socio-demographic characteristics

These included age, sex, religion, ethnicity, self-reported family income class, parent’s education, list of appliances used to access the internet, and perceived relationship with parents, friends and relatives.

### Statistical analysis

Data were entered in EpiData version 3.1, transferred and analyzed using IBM SPSS Statistics for Windows, version 20.0. Descriptive statistics (mean, frequency and percentage) were calculated. The relationship between internet addiction, socio-demographic variables (independent variable) and sleep quality (dependent variable) was assessed using bivariate and multivariable logistic regression analyses. Variables which were significant with sleep quality at p < 0.05 in bivariate logistic regression analyses were modeled in multivariable logistic regression estimating the adjusted odds ratio.

## Results

Table 1 provides the socio-demographic characteristics of the study sample (N = 390). The mean age was 15.0 (SD = 1.0) years and more than half were female (55.4%). In terms of ethnic backgrounds, 47.2% of the participants belonged to the Indigenous ethnicity. Nearly half of the participants (47.4%) identified their family belonging to the upper-middle-income class. It was found that the parents of participants mostly completed bachelor’s level education. Majority of the participant’s main source of family income was business (43.8%). Most participants had access to at least a smartphone (67.4%) and/or laptop (46.2%). A higher proportion of participants reported better self-perceived relationships with parents and friends compared to relatives.

**Table 1:** Socio-demographic characteristics of the adolescent participants.

Socio-demographic characteristics		Frequency	Percentage
**Age**	≤15 years	287	73.6
Mean±SD (range): 15.0±1.0 (13–20) years	>15 years	103	26.4
**Sex**	Male	216	55.4
	Female	174	44.6
**Religion**	Hindu	323	82.8
	Other	67	17.2
**Ethnicity**	Indigenous	184	47.2
	Brahmin	90	23.1
	Chhetri	89	22.8
	Dalit	23	5.9
	Others	4	1.0
**Self-reported family income class**	Upper middle class	185	47.4
	Middle class	163	41.8
	Upper class	42	10.8
**Parent’s education (any parent)**	Bachelor’s and higher	110	28.2
	Secondary	109	27.9
	Primary	76	19.5
	Higher secondary	69	17.7
	Illiterate	26	6.7
**Main source of household income**	Business	171	43.8
	Service	114	29.2
	Agriculture	69	17.7
	Labour	31	7.9
	Others	5	1.3
**Devices for internet use (Multiple responses possible)**	Smartphone	263	67.4
	Laptop	180	46.2
	Tablet	90	23.1
	Other	21	5.4
**Relation with parents**	Good	361	92.6
	Neutral	29	7.4
**Relation with friends**	Good	307	78.7
	Neutral	74	19
	Bad	9	2.3
**Relation with relatives**	Good	199	51.1
	Neutral	176	45.1
	Bad	15	3.8

The mean Internet Addiction Test score was 12.1 (SD = 6.5). Around one-fifth (21.5%) of the participants had borderline internet addiction and 13.3% had possible internet addiction. The mean Pittsburgh Sleep Quality Index score was 4.6 (SD = 2.3), with 31.0% of the participants falling in the poor sleep quality category (Table 2).

**Table 2:** Prevalence of Internet addictions and sleep quality among adolescent participants.

Variables	Frequency	Percentage
Internet Addiction test, mean±SD	12.1±6.5	
**Internet addiction category**		
No internet addiction	254	65.2
Borderline internet addiction	84	21.5
Possible internet addiction	52	13.3
Likely internet addiction	0	0.0
Pittsburgh Sleep Quality Index, mean±SD	4.6±2.3	
**Sleep quality category**		
Good sleep quality	269	69.0
Poor sleep quality	121	31.0

In bivariate and multivariable analyses (Table 3), sleep quality was significantly associated with internet addiction, age, and ethnicity. Adolescents with borderline and possible internet addiction were more likely to experience poor sleep quality compared to no internet addiction. Higher age group participants were found to be associated with poor sleep quality. In terms of ethnicity, the participants from Indigenous and Chhetri caste groups were more probable to experience poor sleep quality in comparison to other groups.

**Table 3:** Bivariate and multivariable analysis of socio-demographic characteristics, internet addictions and poor sleep quality among adolescent participants.

Variables	COR (95% CI)	P-value	AOR (95% CI)	P-value
**Internet addiction**				
No IA	Ref.		Ref.	
Borderline IA	1.85 (1.09–3.13)	0.022*	1.790 (1.034–3.096)	0.037*
Possible IA	3.98 (2.15–7.40)	<0.001*	3.789 (1.984–7.237)	0.000*
**Age**				
≤15 years	Ref.		Ref.	
>15 years	1.81 (1.13–2.90)	0.013*	1.599 (0.965–2.649)	0.068
**Sex**				
Male	Ref.		-	
Female	0.87 (0.56–1.33)	0.5		
**Religion**				
Hindu	Ref.		-	
Other	1.30 (0.75–2.26)	0.352		
**Ethnicity**				
Brahmin	Ref.		Ref.	
Chhetri	2.73 (1.37–5.44)	0.004*	2.823 (1.378–5.787)	0.005*
Indigenous	2.65 (1.43–4.92)	0.002*	2.204 (1.158–4.196)	0.016*
Dalit	0.69 (0.18–2.62)	0.590	0.800 (0.208–3.075)	0.745
Others	4.63 (0.61–35.32)	0.140	3.119 (0.364–26.762)	0.300
**Self-reported family income class**				
Upper middle class	Ref.		-	
Middle class	0.878 (0.56–1.38)	0.575		
Upper class	0.930 (0.45–1.93)	0.847		
**Parent’s education (any parent)**				
Bachelor’s and higher	Ref.		-	
Secondary	0.905 (0.33–2.49)	0.846		
Primary	1.453 (0.56–3.76)	0.442		
Higher secondary	1.448 (0.53–3.93)	0.468		
Illiterate	1.163 (0.45–3.03)	0.757		
**Main source of household income**				
Business	Ref.		-	
Service	0.96 (0.51–1.82)	0.902		
Agriculture	0.83 (0.45–1.51)	0.533		
Labour	0.95 (0.39–2.35)	0.916		
Others	0.50 (0.05–4.73)	0.546		
**Relation with parents**				
Good	Ref.		-	
Neutral	1.00 (0.44–2.27)	0.999		
**Relation with friends**				
Good	Ref.		-	
Neutral	1.17 (0.29–4.77)	0.828		
Bad	1.27 (0.74–2.16)	0.389		
**Relation with relatives**				
Good	Ref.		-	
Neutral	1.70 (0.58–5.00)	0.333		
Bad	1.29 (0.83–1.99)	0.260		
Goodness of fit χ2 (Hosmer-Lemeshow) = df = 7, p = 0.864				

*P<0.05, COR = Crude Odds Ratio, AOR = Adjusted Odds Ratio

## Discussions

This study aimed to assess the prevalence of internet addiction and poor sleep quality among adolescents in a peri-urban setting in Nepal. Borderline internet addiction and possible internet addiction were prevalent in 21.5% and 13.3% respectively. Around one-third (31.0%) were found to experience poor sleep quality. These results corroborate with findings of similar other studies conducted in Nepal [25] and elsewhere [9, 31].

Higher age during adolescence depicted poorer sleep quality compared to younger age groups. In contrast to other studies where girls were more likely to have poor sleep quality than boys [32], this study did not find the association between sleep quality and sex. A statistically significant association was found between internet addiction and poor sleep quality in the present study. The current study findings were supported by research conducted among female adolescents in Taiwan [33] and the high school students in Turkey [34] and Japan [35] with the positive relationship between internet addiction and poor sleep quality. Contrary to this study findings, no association was established between poor sleep quality and internet use in a study conducted among university students in Canada. The young population is often found to surf the internet and use other media to cope with sleep problems [36]. Based on our findings, it was found that as the adolescents got older, their sleep quality worsened as well. Of the studies conducted in the UK, one study found that using phones during bedtime reduced sleep duration by 21 minutes [37] and another study associated 45 minutes of reduced sleep duration with the use of phones at bedtime among pre-teens [38, 39]. However, a study in Canada found a negative association between the presence of phones and sleep duration but no association between phone use and sleep duration [40]. The current study showed around 29% of the participants had slept on an average of 7 hours and 23% had slept 8 hours. Whereas, a study conducted in Korea found that participants slept for 6 hours (26%), 7 hours (31.1%), 8 hours (29%) and 9 hours (13.9%). Excessive internet use was presumed to be the cause of sleep deprivation by displacing sleep time [41].

Awareness of poor sleep quality as a result of increased internet use and screen time should be carried out. Parents and school authorities may be at the central focus of the interventions aiming to reduce internet addiction as well as sleep quality.

The results in this study are reliant on self-reported behavior by the participants and it represents their views and perspectives. There might be a discrepancy between self-reported behavior and actual behavior practice. Negligence and underestimation of their own addictive behaviors might be underrepresented based on self-reported behavior. The study has not covered mental health problems associated with internet addictions. Being a cross-sectional study design, the results of this study does not provide causality, instead provide evidence between internet addiction and poor sleep quality.

The study provides background information on internet addiction and the sleep quality to design large scale studies in different age groups, their cognitive and behavioural development as well as overall health impact.

## Conclusions

The current cross-sectional study demonstrated the one-third prevalence of internet addiction with poor sleep quality in peri-urban settings of Nepal. Ethnic groups such as Chhetri and Indigenous groups were more prone to problems related to internet addiction and poor sleep quality. These socio-demographic variables depict the complex relationships and influence over the problems of adolescents. The findings also suggest that there are significant associations between internet addiction and sleep quality. The findings that adolescents with internet addiction are more likely to experience poor sleep quality supported the relevancy of this topic in recent times. It would benefit future studies to emphasize and dig deeper into the underlying causes and patterns of these problems. The findings have implications for adolescents, parents, school authorities and researchers.

## Abbreviations

DSM-IV: Diagnostic and Statistical Manual of Mental Disorders, 4th edition
DSM-V: Diagnostic and Statistical Manual of Mental Disorders, 5th edition
IA: Internet Addiction
IAS: Internet Addiction Scale
IAT: Internet Addiction Test
PSQI: Pittsburg Sleep Quality Index
